# Lose the Hardware: The Rise of Leadless Modular Cardiac Pacing

**DOI:** 10.3390/jcm15207806

**Published:** 2026-10-10

**Authors:** Mohamad Al-Mir, Mikhael F. El-Chami

**Affiliations:** Division of Cardiology-Section of Electrophysiology, Emory University School of Medicine, Atlanta, GA 30322, USA

**Keywords:** leadless pacemaker, modular pacing, atrioventricular synchrony, standalone leadless pacemaker, dual-chamber leadless pacemaker

## Abstract

Leadless pacemakers have reshaped the approach to cardiac pacing by eliminating transvenous leads and subcutaneous pockets. Since the introduction of the first leadless pacemakers, leadless pacing has advanced from single-chamber ventricular systems to atrioventricular synchronous pacing, standalone atrial pacing, and fully leadless dual-chamber platforms, enabling patient-tailored pacing therapy. The modular leadless pacing concept allows a standalone leadless pacing device to be implanted initially, with additional leadless devices added sequentially as pacing requirements evolve, minimizing unnecessary hardware while preserving the option for future pacing upgrades. This modular approach has expanded to include integration with subcutaneous implantable cardioverter-defibrillators and leadless cardiac resynchronization therapy, moving towards a fully leadless cardiac rhythm management ecosystem. This review summarizes the evolution of leadless pacing, compares currently available devices and their clinical applications, explains leadless pacemaker implantation and end-of-life management, and explores the modular leadless pacing concept as the foundation for future fully leadless cardiac rhythm management.

## 1. Introduction

### 1.1. Evolution of Cardiac Pacing

**Figure 1:**
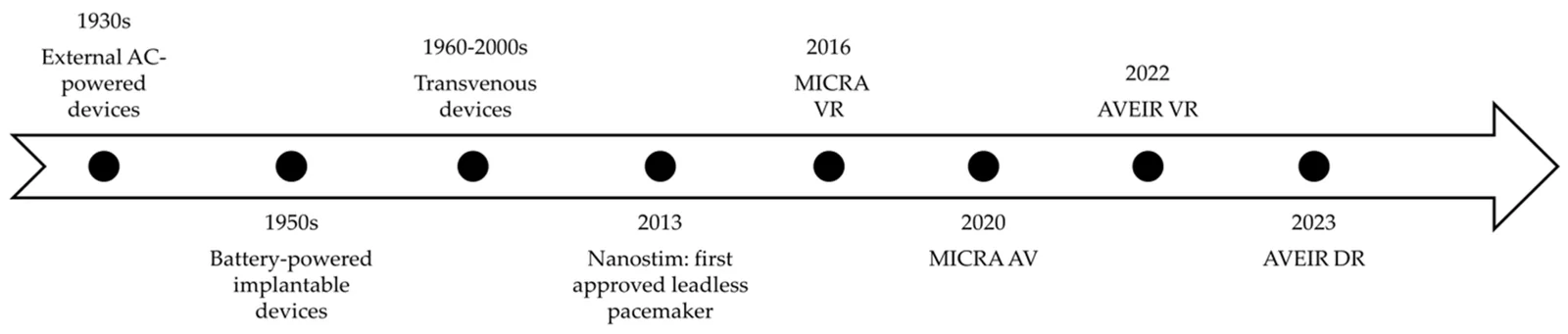
Evolution of Cardiac Pacemakers.

The concept of cardiac pacing originated in the 1930s with the development of the first external devices capable of delivering impulses to the heart via a needle electrode [1]. The field advanced rapidly over the following decades, culminating in the first successful permanent battery-powered pacemaker implantation in a human in 1958 [1,2]. Later innovations, including rate-responsive, multi-chamber pacing and internet-based remote monitoring, expanded the clinical applications of pacemaker therapy while progressively reducing device size (Figure 1) [1,3,4]. Despite these advances, the fundamental architecture of pacing systems had remained largely unchanged, consisting of an extravascular pulse generator linked to one or more transvenous leads threaded through the venous system into the heart [1,4].

### 1.2. Limitations of Transvenous Pacemakers

Transvenous pacemakers (TV-PM) carry a substantial short- and long-term complication burden, stemming primarily from two areas of vulnerability: the transvenous lead(s) and the subcutaneous pocket [4,5]. The MOST trial reported a 4.8% overall complication rate at 30 days after TV-PM implantation [5,6], whereas the FOLLOWPACE study reported a cumulative complication rate of 12.4% within 60 days, increasing to nearly 20% at 5 years [5,7]. Clinically, these complications range from acute events such as lead dislodgement, pneumothorax, vascular injury, and pocket hematoma to chronic device-related issues including lead fractures, insulation failures, venous occlusion, tricuspid valve regurgitation (TR), and infections [5,8]. In addition to device-related complications, transvenous lead extraction (TLE) is not uncommonly required during follow-up due to lead-related failure, need for an upgrade in the context of an occluded vein, and, most importantly infection [5,8]. Approximately 5–10% of patients undergoing TV-PM implantation experience peri-procedural complications, while serious extraction-related adverse events, including death, may occur in 1.4–5.6% of cases [5,8]. Collectively, these short and long-term risks associated with TV-PM highlight the need for alternative pacing strategies that eliminate leads and subcutaneous pockets [4].

### 1.3. Emergence of Leadless Pacing

As the global population ages and indications for pacing expand, the number of pacemaker implantations continues to rise [4]. In the United States alone, approximately 250,000 pacemakers are implanted each year [9]. This growing demand has amplified the potential burden of TV-PM-related complications and the need for less invasive pacing technologies [3,4]. Leadless pacemakers (LP) were conceived to circumvent the complications inherent to TV-PM [4]. By encapsulating the entire pacing system within a single, self-contained intracardiac device, LPs have arisen as a fundamental departure from the traditional architecture of cardiac pacing [3,8].

## 2. Evolution of Leadless Pacemakers

### 2.1. Nanostim (St. Jude Medical/Abbott)

The Nanostim was the first LP to enter the market in 2013 [4]. It employed an active-fixation helix mechanism and was delivered via a transfemoral approach for single-chamber ventricular pacing [3]. In the pivotal LEADLESS II trial (n = 526), implantation success was 95.8% [10]. However, post-market surveillance revealed premature battery failure in 34 of 1423 implanted devices worldwide, with a mean time to failure of 2.9 years [11]. A subsequent single-center analysis of consecutive patients who underwent Nanostim implantation revealed device failure rates up to 37% during a 3-year follow-up period [12].

Due to these safety and reliability concerns, a medical device advisory was issued in 2016, and the Nanostim was ultimately withdrawn from the market, with discontinuation of further implantations [11]. Abbott later redesigned their device and developed the Aveir LP, which received FDA approval in 2022 [4].

### 2.2. MICRA (Medtronic)

The MICRA LP is a tine-based device introduced in 2016 designed for passive fixation within the right ventricle (RV) [13]. In the Micra IDE trial (n = 725), implantation success was 99.2% [13]. At 6 months, the major complication rate was 4%, with complications primarily consisting of cardiac perforation and vascular access-related events [13]. Notably, no device-related infections were reported in the study cohort [13,14].

Longer-term data from the Micra Post-Approval Registry (PAR) reinforced the findings of the IDE trial, demonstrating a 12-month major complication rate of 2.7% [15]. This represented a 63% reduction compared with historical TV-PM cohorts [15]. A 60-month follow-up analysis later revealed a major complication rate of 4.5%, significantly lower than that observed with TV-PMs (8.5%) [16]. Compared to TV-PMs, MICRA device revisions were fewer and unrelated to infection [16]. Real-world comparative evidence from the Micra Coverage with Evidence Development (CED) study, which analyzed Medicare claims data, further supported these observations and found that leadless pacing was associated with 32% fewer chronic complications, 41% fewer device reinterventions, and significantly lower infection rates at 3 years compared with transvenous pacing [17].

To address the limitation of single-chamber ventricular pacing, the MICRA AV LP was later introduced. It utilizes an accelerometer-based algorithm that mechanically detects atrial contractions to achieve atrioventricular (AV) synchrony (Table 1) [3,4]. The MARVEL2 study demonstrated that the MICRA AV improved AV synchrony to 89.2% in a resting state [18]. In the ACCELAV study (n = 152), patients with complete AV block and normal sinus rhythm achieved a mean resting AV synchrony of 85.4% and ambulatory AV synchrony of 74.8% at 1 month [19]. Ambulatory AV synchrony significantly improved to 82.6% after device optimization in a subset of patients [19]. However, AV synchrony progressively declined at heart rates above 80 bpm [3,19]. The upper tracking rates of MICRA AV and MICRA AV2 are limited to 115 and 135 bpm, respectively [3], beyond which the devices transition to a non-tracking rate-responsive mode (VVIR) [20]. Consequently, MICRA AV may be less suitable for younger or more physically active patients who require consistent AV synchrony during periods of exertion and elevated heart rates [3,4,20].

### 2.3. AVEIR (Abbott)

The Nanostim redesign led to the development of the Aveir VR LP, incorporating an active helix-fixation mechanism, improved battery longevity, and an improved docking mechanism to facilitate both implantation and retrieval [3,4]. In the LEADLESS II Phase 2 trial (n = 210), implantation success was 97.6%, with 93.2% freedom from device-related complications at 12 months and an estimated battery longevity of 17.6 years [4,21]. Of the reported adverse events, 73% occurred within the first 3 days after implantation, mainly due to cardiac perforation and premature device deployment [4,21]. The Aveir VR CED study later revealed favorable real-world outcomes compared with TV-PM, including a 30% lower overall complication rate, a 47% lower device-related complication rate, and 61% fewer device reinterventions at 12 months [22]. A post-market analysis of the FDA Manufacturer and User Facility Device Experience (MAUDE) database identified helix-related failures and cardiac perforation-related events among the most frequently reported serious complications with Aveir VR, along with high pacing thresholds and loss of capture [23].

To overcome the limitations of single-chamber ventricular pacing, the Aveir platform has expanded to include the Aveir AR, a standalone atrial LP, and the Aveir DR, the first commercially available dual-chamber LP [3,4]. The Aveir DR LP is composed of separate atrial and ventricular LPs that communicate via proprietary implant-to-implant (i2i) conductive communication [3,24,25]. This system coordinates beat-to-beat AV synchrony using subthreshold wireless signals transmitted through blood and intracardiac tissue [3,25].

In the pivotal Aveir DR study, implantation success was 98.3% [24]. At 3 months, 90.3% of patients were free from serious adverse events [24], and the complication-free rate at 12 months was 88.6% (Table 1) [26]. Complications included cardiac perforation and atrial lead dislodgement, with the latter occurring more frequently within 3 months with the Aveir DR system than what has been reported for conventional dual-chamber TV-PM (3.3% vs. 1.9%) [3,24]. Subsequent CED data showed a 41% lower hazard of overall chronic complications, a 52% lower hazard of device-related complications, and a 51% lower hazard of device reinterventions at 6 months compared to dual-chamber TV-PMs [27]. Despite these favorable outcomes, the rate of cardiac perforation remained higher with the Aveir DR LP than with TV-PMs (1.2% vs. 0.6%) [27].

## 3. Clinical Applications and Patient Selection for Leadless Pacing

Although the introduction of atrial sensing and dual-chamber LPs has expanded potential indications, several patient populations require special clinical considerations.

### 3.1. Patients at High Risk of Infection

Transvenous cardiac implantable electronic device (CIED) infections occur in 1–2% of patients [28], and they carry an in-hospital mortality of 3–11% [29], with 12-month mortality reaching 15–30% [29]. Transvenous CIEDs typically require complete extraction once infected [29,30]. In contrast, large clinical studies including Micra IDE, Micra PAR, and Aveir LEADLESS II Phase 2 studies have reported no LP-related infections among more than 3000 implanted patients [30]. Similarly, the Micra CED study revealed an infection rate below 0.2% at 3 years [17], with no device infections observed among patients who developed bacteremia with a Micra LP in situ [14]. These findings support the use of LPs in populations at elevated infectious risk, including patients on hemodialysis, immunosuppressed patients, those with uncontrolled diabetes mellitus, and patients with a history of device infection or infective endocarditis [4,30]. The reduced infection risk is multifactorial and is attributed to the elimination of a subcutaneous pocket, reduced device handling during implantation, the small intravascular device surface area, progressive device endothelialization and encapsulation, and the parylene coating of the device titanium casing, which may inhibit biofilm formation [31].

### 3.2. Venous Access Limitations

Beyond mitigating infection risk, LPs also offer advantages in patients with limited or occluded upper-extremity venous access, particularly those with end-stage renal disease, as the femoral delivery route bypasses the upper venous system [3,4]. The Micra has demonstrated an acceptable safety profile in hemodialysis patients, with complication rates comparable to non-dialysis patients [32].

### 3.3. Tricuspid Valve Interventions and Tricuspid Regurgitation

Patients undergoing tricuspid valve repair are at higher risk of postoperative conduction disorders requiring permanent pacing because of the anatomical proximity between the AV node and the anteroseptal commissure of the tricuspid valve [4,33]. In these patients, LPs may be beneficial because they eliminate the transvalvular leads, which are associated with an increased risk of TR development and progression [4,34,35]. Although concerns remain regarding potential LP-tricuspid valve interaction, systematic evidence suggests a low incidence of device-related tricuspid dysfunction [36]. In a systematic review of 272 patients with prior surgical or transcatheter tricuspid valve interventions, implantation success was 99.3%, with rare device–valve interactions and no worsening of tricuspid regurgitation attributable to the LP or its delivery system [37]. These results support LPs as an attractive valve-sparing pacing strategy, particularly when implanted at the base of the RV away from the tricuspid apparatus [4]. Shorter LPs might be more advantageous in this setting because they are less likely to interfere with the TV apparatus.

### 3.4. Inferior Vena Cava Filters and Internal Jugular Access

**Figure 2:**
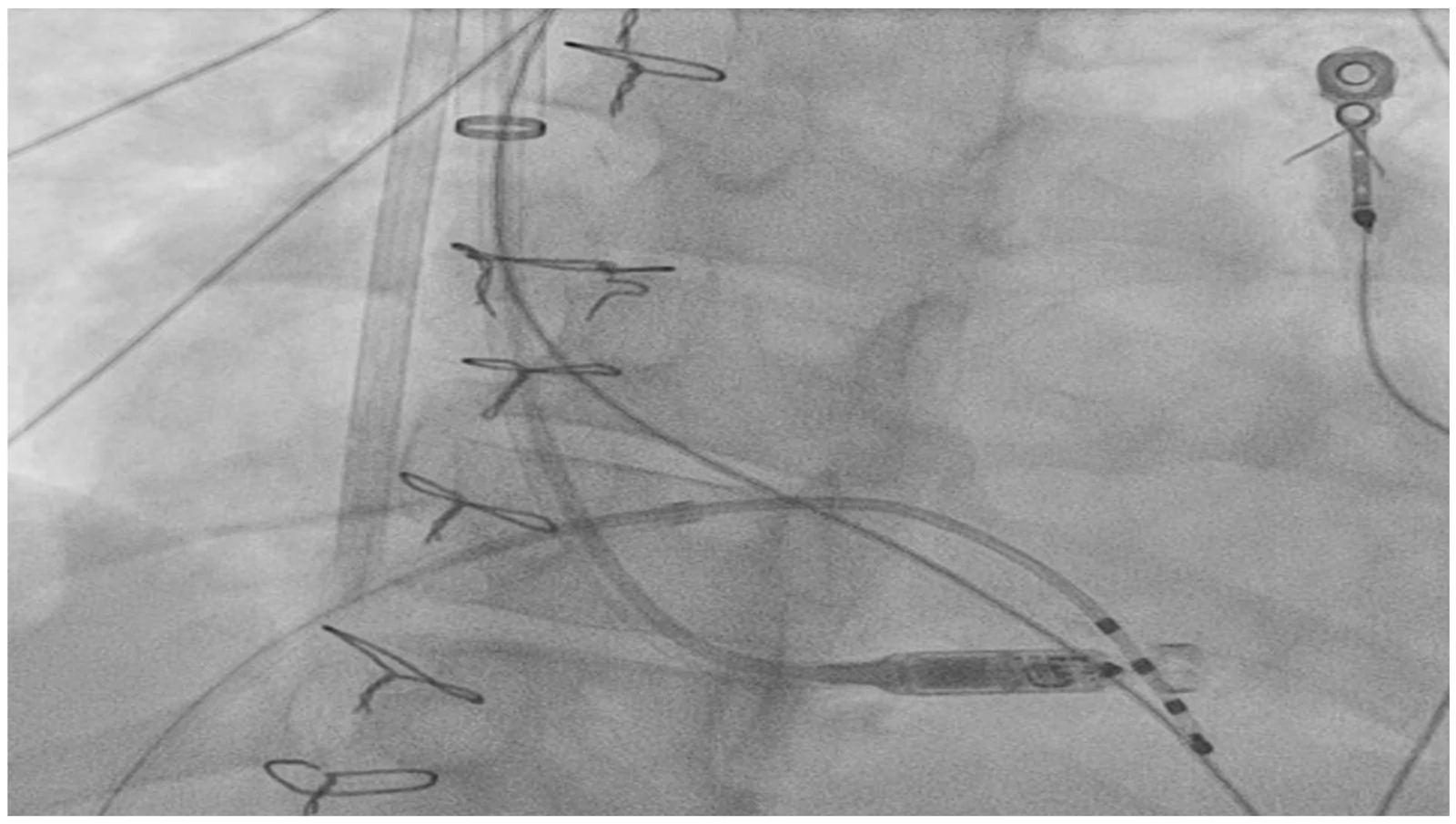
A Micra LP Implanted Via the IJ Route.

The presence of an inferior vena cava (IVC) filter is no longer considered an absolute contraindication to LP implantation [4,38]. In a multicenter series of 22 patients, implantation through IVC filters was successful in 95.5% of cases [39]. In patients with recently implanted or occluded filters, alternative access via the internal jugular vein can be safely used, allowing the delivery sheath to be advanced through the superior vena cava and across the tricuspid valve for septal implantation (Figure 2) [3,4,38]. In such cases, internal jugular (IJ) access is preferred over femoral access [4]. The IJ access is gaining traction as an alternative to the traditional femoral route. The advantage is a straight route to the right atrium (RA) and easier access to the interventricular septum. In a study by Molitor et al., 100 patients who underwent LP implantation via the IJ route were compared to a matched cohort of 100 patients implanted via the traditional femoral route [40]. IJ access was associated with lower procedural time and complications [40]. This access may be advantageous in patients with extreme right-sided chamber sizes (small or large RA/RV), patients in whom the IVC enters the RA at an extreme angle, and patients who have undergone transcatheter tricuspid valve replacement [41,42].

## 4. Implantation of Leadless Pacemakers

LP implantation is typically performed percutaneously via the right femoral vein under fluoroscopic guidance, using either moderate sedation or general anesthesia. In patients on oral anticoagulation, LP implantation can be safely performed without interrupting therapy, as it has been shown to be safe without an increased risk of perforation or vascular adverse events regardless of anticoagulation regimen [43].

Following ultrasound-guided venous access and serial dilation, a large-bore introducer sheath is inserted (inner diameter: 23 Fr for Micra and 25 Fr for Aveir; outer diameter: 27 Fr for both systems) [3,44,45]. The delivery catheter is then advanced through the IVC, across the tricuspid valve, and positioned against the RV septum.

The two commercially available systems differ in their fixation mechanisms and implant techniques. The Micra (25.9 × 6.7 mm) employs four self-expanding nitinol tines for passive fixation [13,44]. The device is deployed by applying gentle forward pressure to create a “gooseneck” deformity on fluoroscopy, followed by controlled manual retraction of the delivery cup to allow engagement of at least two orthogonal tines within the myocardium [38,44]. Because the tines are structurally separate from the pacing electrode, tissue contact is spatially isolated from the sensing and pacing interfaces [44,46]. The smaller device profile minimizes interaction with the tricuspid valve apparatus, thereby permitting Micra mid-septal positioning [3].

**Figure 3:**
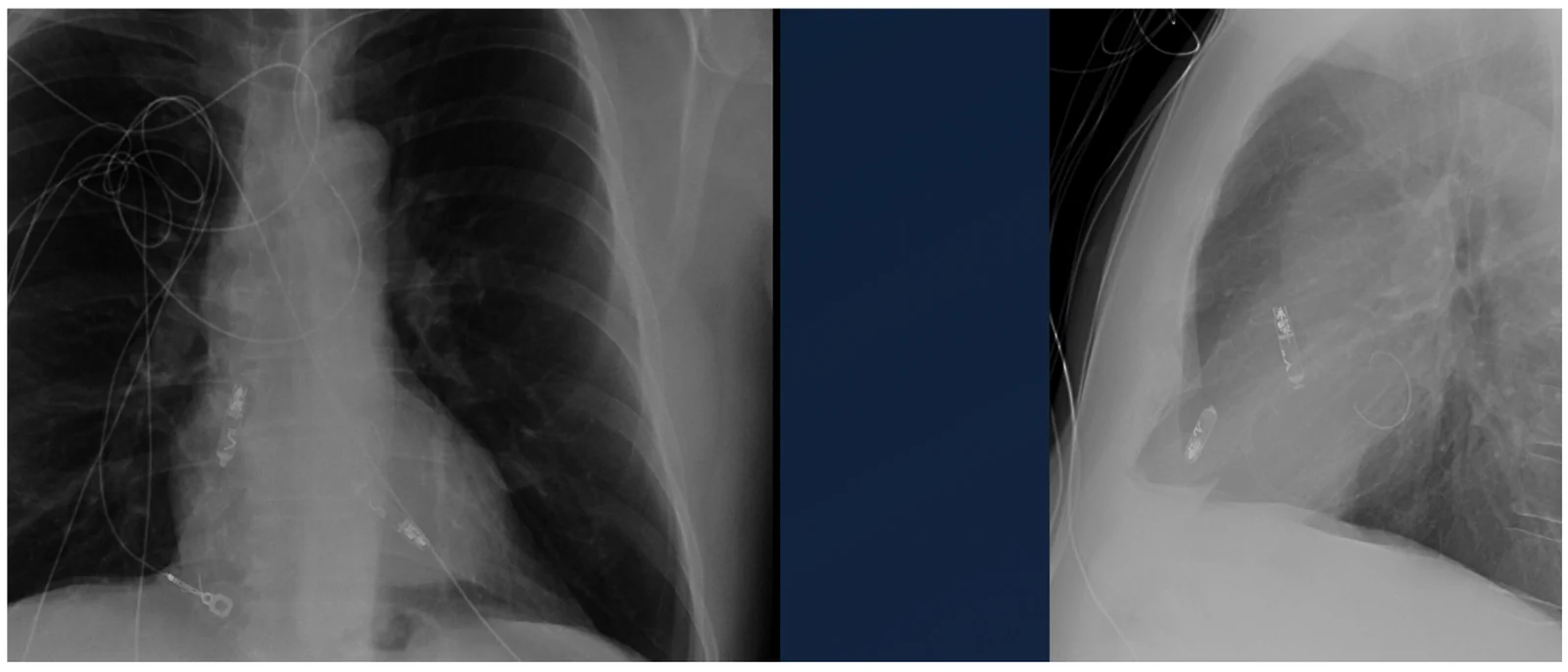
PA & Lateral Chest X-Ray Views after Aveir DR Implantation.

In contrast, the Aveir (38 × 6.44 mm) utilizes an active fixation helix that is screwed into the myocardium [3,45]. It is deployed using controlled clockwise rotations of a radiopaque marker under fluoroscopy [45]. An important advantage of this design is the ability to perform electrical mapping prefixation [3]. Because the electrode remains exposed during positioning, pacing threshold, R-wave amplitude, and impedance can be assessed before deployment, reducing the need for repositioning [3,45]. In the Aveir IDE study, this approach resulted in no repositioning in approximately 83% of implants [47]. However, the larger device profile may make implantation more challenging in smaller or more vertically oriented RVs [45]. It also requires more distal septal positioning to avoid interaction between the proximal docking button and the tricuspid valve [45]. For Micra and Aveir, septal implantation is essential, and using orthogonal X-Ray views and contrast to confirm the site of implantation might help mitigate the risk of perforation (Figure 3). For atrial implantation, the ostium of the right atrial appendage is the favored target [24], as this location optimizes device-to-device communication and is associated with fewer postprocedural dislodgements compared to mid or distal appendage sites [3,24]. However, thinner atrial myocardium may increase the technical complexity of fixation [3].

After deployment, contrast injections are used to confirm wall contact and positioning, regardless of device type [3,4]. Adequate myocardial engagement is confirmed by three electrical parameters: pacing capture threshold (PCT), R-wave amplitude, and impedance. Both systems generally target a PCT ≤ 1.5 V at 0.4 ms and R-wave amplitude ≥ 5 mV, with impedance > 400 Ω suggesting stable tissue contact [4,48,49]. Thresholds often improve over the first several minutes after implantation, particularly with Aveir, which may exhibit a transient increase in PCT that typically stabilizes to ≤1 V within 5–10 min [4,45]. Notably, pre-fixation PCT appears to be a stronger predictor for chronic performance than the immediate post-fixation value [49]. A final PCT > 2.0 V is generally not acceptable for either system [3,4]. Operators must balance the benefits of repositioning against procedural risk, particularly in patients at high risk of perforation, as device repositioning itself is an independent risk factor for cardiac perforation [4,50].

### Operator Learning Curve

The operator learning curve plays a key role in procedural safety and efficiency. Early experience with Nanostim showed significantly lower complication rates among operators who had performed more than 10 implants, and structured training programs in later leadless pacing studies were associated with substantial reductions in perforation and dislodgement events [3]. Similarly, the Micra PAR reported a decline in pericardial effusion rates from 1.5% in the IDE trial to 0.4%, attributed in part to increased operator experience and greater adoption of a septal implantation approach [3]. A bi-center analysis of 111 Micra implantations further confirmed that procedural efficiency improves significantly with experience, with reductions in procedure time, fluoroscopy duration, and time to first deployment observed with increasing case volume [51]. For the Aveir system, procedural efficiency generally stabilizes after 3–4 implants, reflecting a favorable learning curve [52]. However, fluoroscopy and procedure times remain longer than with Micra, largely due to continuous fluoroscopic guidance during helix deployment and additional system setup steps [52,53]. Interestingly, operators in the Aveir DR pivotal trial had not attained the learning curve for atrial LP implantation despite prior ventricular LP experience, which may have contributed to the higher-than-expected atrial LP dislodgement rate (3.3%) [3,24]. This evidence stresses the importance of structured training programs, proctored initial cases, and maintenance of case volume for both systems.

## 5. End-of-Life Management of Leadless Pacemakers

End-of-life (EOL) management remains one of the most important unsolved challenges in leadless pacing. Estimated device longevity varies according to device type and programming settings. The original Micra VR has a projected longevity of approximately 11.8 years based on 5-year performance data [3,16]. In contrast, the newer generation Micra VR2 and Aveir VR batteries are estimated to last approximately 16.7 and 17.6 years, respectively [3,21]. At the time of battery depletion, two primary management strategies exist: device retrieval or abandonment [54]. The choice of strategy ultimately depends on the patient’s pacing requirements and clinical needs [54].

**Figure 4:**
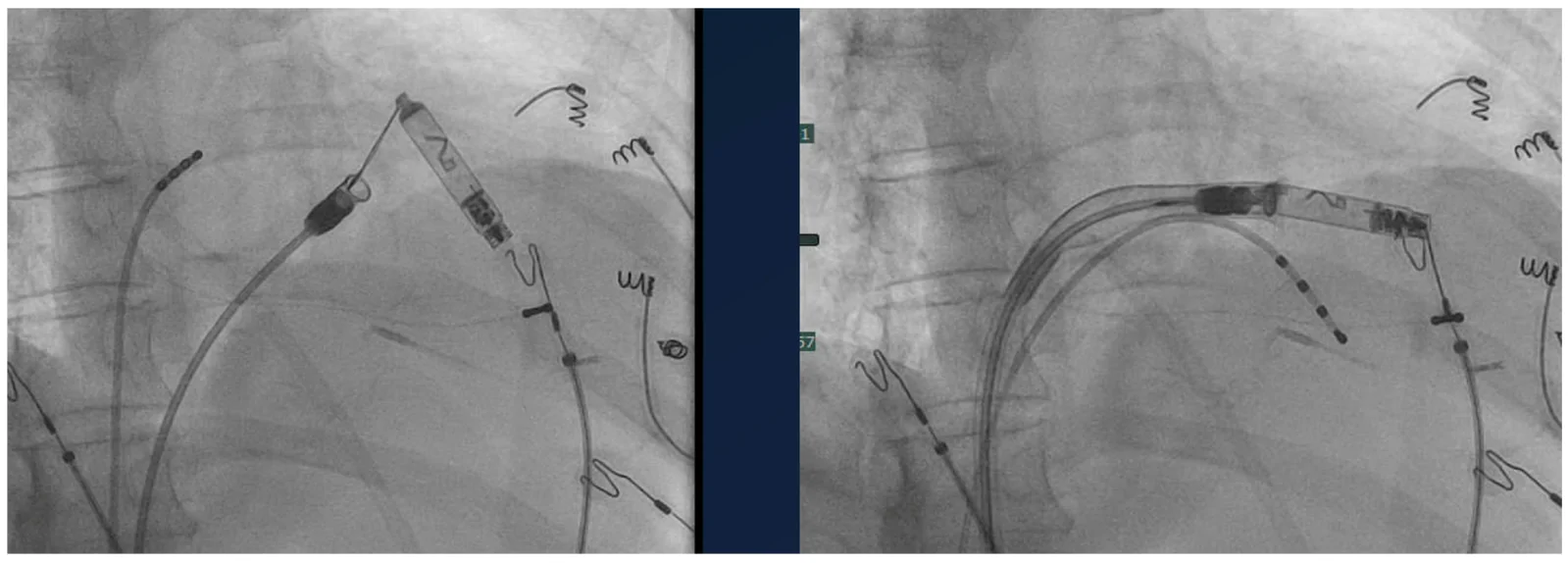
Extraction of an Aveir VR.

The Aveir system is specifically engineered to facilitate chronic retrieval. Its active helix fixation allows engagement of a dedicated proximal docking feature using a retrieval catheter, followed by counterclockwise rotation to unscrew the helix from the endocardium (Figure 4) [45]. Reported chronic retrieval success rates are approximately 87.6% and 85% at mean follow-ups of 2.6 and 4 years after implantation, respectively [55,56].

**Figure 5:**
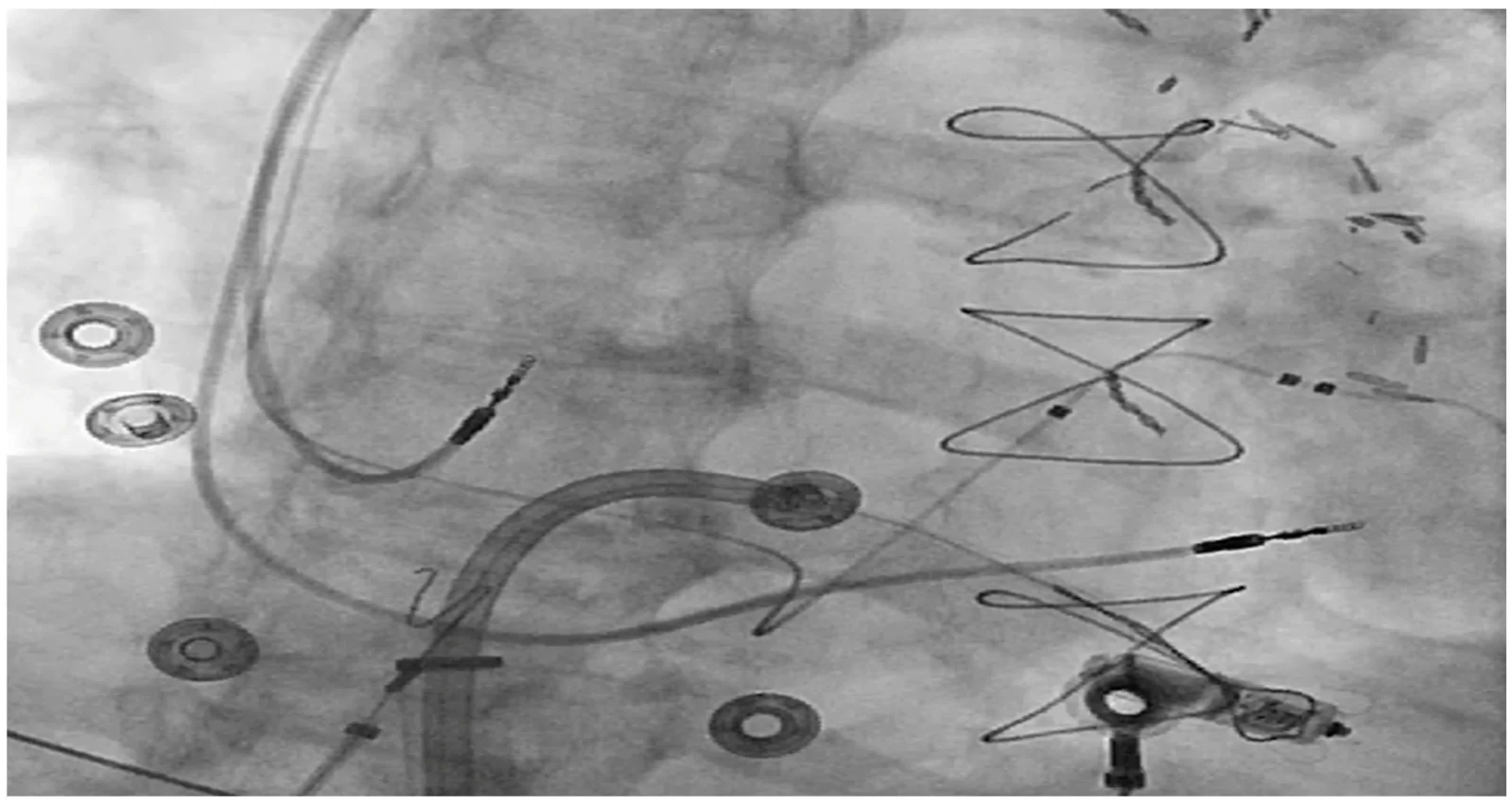
Extraction of a Micra VR.

Micra retrieval relies on a snare-based technique. After inserting a steerable sheath, a single or triple-loop snare is advanced into the RV to capture the device’s proximal retrieval feature (Figure 5) [57]. Controlled traction is then applied to collapse the nitinol tines, allowing disengagement and withdrawal of the device into the introducer sheath [57]. Results from a recent multicenter analysis of 85 patients across 11 centers in Europe and the United States demonstrated a 96.5% success rate for Micra device retrieval using single or triple-loop snares, with a median device dwell time of 96 days [58]. There were five procedural complications, two attributable to cardiac perforation and three device embolizations [58]. Importantly, this study revealed that apical implantation and longer dwell time were significant predictors of unsuccessful device removal, with the odds of removal failure increasing by 40% for every additional 180 days the device remained implanted [58]. Longer-term outcomes were reported in a single-center series, which demonstrated an 88.9% retrieval success rate after a mean implant duration of 3.1 years, with no procedure-related adverse events [59]. One failure occurred in a device that was extensively embedded in ventricular tissue [59]. In this series, 7 and 9 year-old devices were extracted successfully [59].

Across both Micra and Aveir systems, progressive device endothelialization and encapsulation may increase retrieval complexity over time [4,31]. When retrieval is not feasible or not pursued, device abandonment is an alternative strategy. In this approach, the depleted LP is programmed to a nonfunctional mode (OOO), and a new LP is implanted adjacent to the existing device. There have been no reported interactions between abandoned and replacement devices, although one case report demonstrated ventricular oversensing by a newly implanted LP caused by an abandoned LP [3,60]. Experimental cadaveric data suggest that the RV can accommodate up to 3 Micra devices without direct physical contact [61]. Among nearly 1800 patients followed for 5 years in the Micra PAR, 83 required an alternative pacing device, with 11 patients undergoing Micra extraction and 72 managed by abandonment [16]. However, long-term clinical data on cumulative device burden, electrical interference, and potential impact on ventricular function remain limited, and the 2025 Appropriate Use Criteria highlight device replacement complexity as a major limitation of leadless pacing, especially in younger patients who may require ≥3 devices over their lifetime [62].

## 6. The Modular Leadless Pacing Concept

### 6.1. Overcoming Single Chamber-LP and Achieving AV Synchrony

Early LPs were confined to single-chamber ventricular pacing, restricting their use primarily to patients with permanent atrial fibrillation and AV block or slow ventricular response, as well as those with transient sinus arrest and low expected ventricular pacing requirement (<5%) [4,63]. Overall, only a minority of patients with pacing indications (~20%) could be considered for early leadless systems due to the absence of atrial pacing and loss of AV synchrony [4,24].

**Figure 6:**
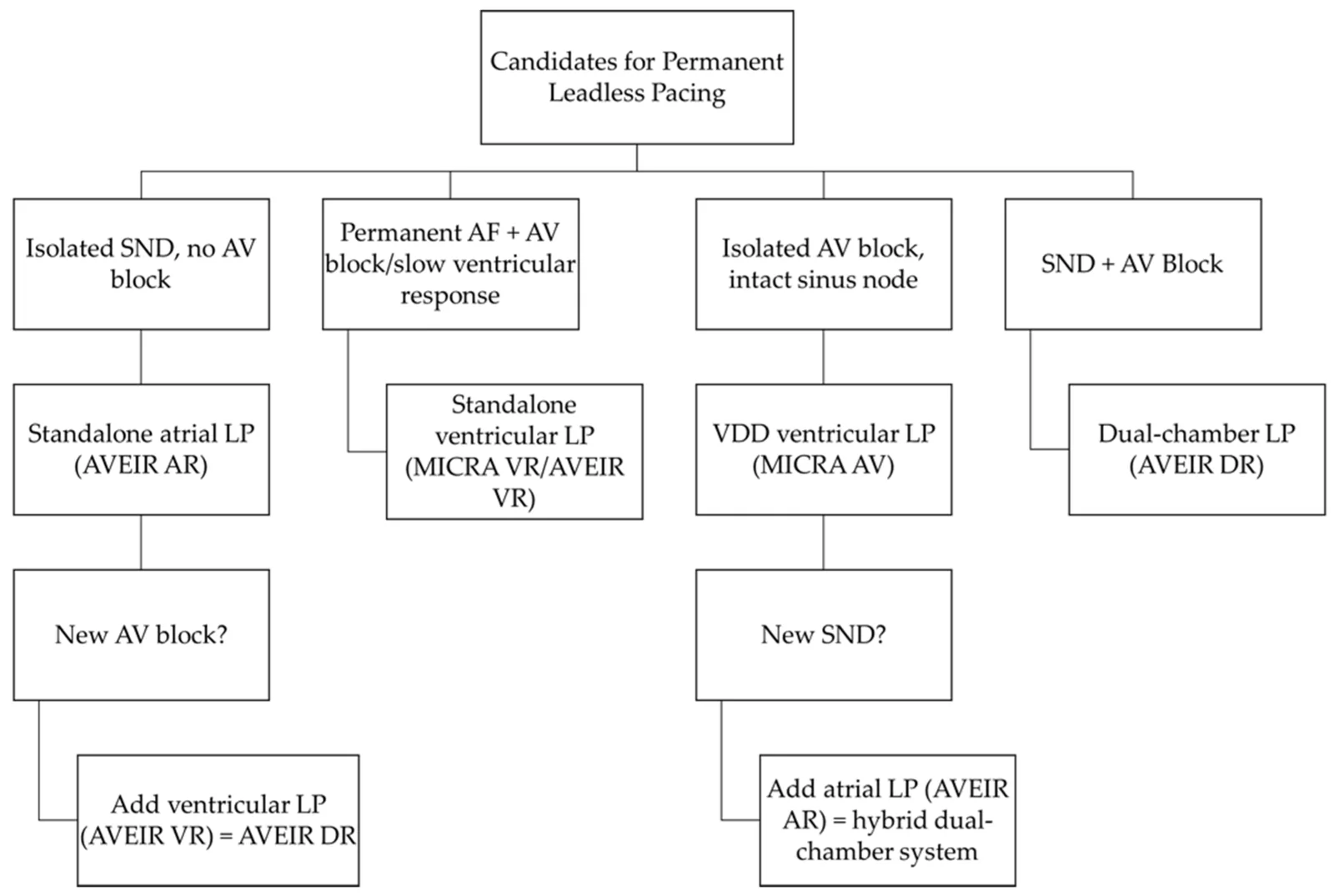
Modular Leadless Pacing: A Building-Block Framework for Tailored Rhythm Management. SND = Sinus Node Dysfunction, AF = Atrial Fibrillation.

The modular pacing concept tackles this limitation through a “building-block” approach to cardiac pacing, in which independently functioning devices are combined as needed to construct an inter-communicating pacing system. This strategy enables restoration of AV synchrony while retaining a low intracardiac hardware burden by allowing initial standalone single-chamber implants with the option for future pacing system expansion (Figure 6). In doing so, it may reduce the aggregate complication burden associated with multiple devices, such as device dislodgement and perforation [4].

### 6.2. Standalone Atrial Pacing and System Upgradability

The Aveir AR system provides standalone atrial leadless pacing for patients with sinus node dysfunction without AV block, a population that has traditionally received dual-chamber TV-PMs despite needing only atrial support [64]. In the Leadless ARRIVE study, Aveir AR demonstrated comparable mortality with fewer 1-year overall complications compared with right atrial and dual-chamber TV-PMs, with reduced device-related complications and fewer reinterventions across both comparisons [64]. These findings emphasize the benefits of targeted standalone atrial leadless pacing in appropriately selected patients.

A key advantage of a modular pacing platform lies in its upgradability. A single-chamber leadless device can be upgraded to a dual-chamber system by implanting a second LP, allowing tailored pacing escalation in patients whose requirements may evolve without replacing existing hardware [4,65,66,67]. This upgradability has already been demonstrated in clinical practice. An 87-year-old male patient on hemodialysis with a pre-existing Micra AV developed symptomatic sinus node dysfunction and subsequently underwent successful implantation of an Aveir AR LP [65]. This created a hybrid dual–chamber leadless pacing system composed of devices from different manufacturers. Rather than pursuing transvenous pacing, which was undesirable given the patient’s hemodialysis status and vascular access concerns, an Aveir AR was implanted to provide atrial pacing [65]. Atrial contractions generated by the Aveir AR were sensed by the Micra AV accelerometer, resulting in coordinated ventricular pacing with approximately 89% synchrony [65]. A key insight is that the two devices did not require i2i communication. Instead, inter-device coordination occurred through a physiologic mechanism, demonstrating cross-manufacturer LP integration. However, this system inherited all limitations of Micra AV mechanical sensing, including reduced AV synchrony at higher heart rates, upper tracking rate limitations, and sensitivity to body motion artifacts [3,19,20].

Prospective evidence supporting modular LP upgradability comes from the multicenter study by Bongiorni et al., which evaluated upgrading single-chamber ventricular LPs to dual-chamber systems [66]. Among 35 patients with a pre-existing Aveir VR who underwent implantation of an atrial LP, successful pairing was achieved in 91.4% of cases [66]. The overall complication rate was 14.3%, with all complications being procedure-related, likely reflecting the early experience with an atrial LP implantation [66]. These outcomes support the modular strategy in which single-chamber leadless pacing is initially selected when appropriate, with the option of future system expansion if pacing needs evolve, thereby avoiding the upfront risks, battery drain, and hardware burden associated with unnecessary dual chamber pacing [4,66].

We reported our institutional experience with modular pacing at Emory Healthcare in a retrospective study of 89 patients who underwent AVEIR LP implantation between 2022 and 2025 [67]. Patients with isolated sinus node dysfunction received standalone atrial LPs, whereas those with isolated AV block received standalone ventricular LPs. Dual-chamber systems were reserved for patients requiring dual-chamber pacing. The overall complication rate was 3.4% [67]. One patient with an atrial LP developed atrial fibrillation with slow ventricular response and subsequently required implantation of a ventricular device 10 months later, illustrating the feasibility of staged modular escalation [67].

Battery longevity remains an important challenge for modular LP systems, especially in dual-device configurations requiring continuous i2i communication. The Aveir DR i2i study revealed that i2i communication substantially impacts battery longevity, with projected lifespans of 5.3 years for atrial LPs and 9.9 years for ventricular LPs in DDD(R) or DDI(R) mode at 12 months [68]. Higher pacing outputs, faster base rates, higher pacing frequency, and elevated i2i communication settings were all significantly correlated with reduced battery longevity [68]. Although lowering the i2i communication setting preserves battery life, it may compromise system performance; therefore, a balance must be struck between maintaining reliable i2i communication and maximizing battery longevity [3,68,69].

To address this issue, an AAI(R) + VVI programming mode was recently introduced for the Aveir DR system in patients with sinus node dysfunction, intact AV conduction, and a low ventricular pacing burden [70]. This i2i communication-free dual-chamber mode allows each LP to function independently. The atrial LP paces the atrium autonomously, while the ventricular LP works primarily as a backup or safety-net device [70]. This strategy has been shown to improve projected longevity, extending atrial LP lifespan by 93% (5.8–11.1 years) and ventricular LP lifespan by 61% (10.9–16.9 years) compared with DDD(R) mode at 6 months of follow-up [70]. Unnecessary ventricular pacing was also reduced from 13.9% to 1.5% [70], potentially decreasing the risk of pacing-induced atrial fibrillation and heart failure hospitalizations [71].

A fundamentally different approach to dual-chamber leadless pacing has been recently demonstrated in a preclinical ovine model by Piccini et al. Rather than relying on two separate communicating devices, investigators implanted a single LP within the triangle of Koch to achieve simultaneous atrial and ventricular sensing and pacing [72].

In 12 sheep, implantation was successful in all cases, and no complications occurred during 6 months of follow-up [72]. The device reliably sensed both native atrial and ventricular activity, and it maintained stable electrical parameters, including atrial capture thresholds of 0.6 ± 0.2 V at 0.24 ms and ventricular capture thresholds of 1.2 ± 0.2 V at 0.24 ms [72]. Device retrieval was successful in all 6 attempted cases, and reimplantation was successful in 5 of those attempts, with immediate restoration of dual-chamber pacing on the first attempt [72]. This approach is promising from a life-cycle management perspective because it eliminates the need for i2i communication, potentially preserving battery longevity and most importantly reducing hardware burden.

However, translation to humans remains challenging. The anatomy of the triangle of Koch varies substantially in humans with respect to heart size, age, and AV nodal location [73,74]. The position of the His bundle can vary considerably as well, sometimes occupying superior and other times inferior portions of the triangle [74]. This variability may make it difficult to consistently achieve reliable atrial and ventricular pacing from a single device, especially in patients with structural heart issues, atrial enlargement, or conduction system fibrosis [72,75]. The anatomical proximity of the AV node and His bundle raises concerns about potential inadvertent injury to the conduction system and iatrogenic heart block during implantation [73,74].

### 6.3. The Modular Cardiac Rhythm Management System

Subcutaneous Implantable Cardioverter-Defibrillator (S-ICDs) provide vasculature-sparing defibrillator therapy but lack endocardial pacing capabilities, limiting their use in patients who require antitachycardia pacing (ATP) [3]. The modular Cardiac Rhythm Management (mCRM) system (Boston Scientific) addresses this limitation by combining the EMBLEM S-ICD with the EMPOWER LP. In this system, the S-ICD detects ventricular tachyarrhythmias and wirelessly communicates with the LP, which subsequently delivers ATP [76]. The Modular ATP trial evaluated the safety and efficacy of this system. At 6 months, wireless device-device communication success rate was 98.8%, and 97.4% of patients had adequate pacing thresholds [76]. At 1-year follow-up, 97.2% of patients remained free from LP-related major complications, and 88.5% were free from overall mCRM system-related complications [77]. ATP delivery was successful in 67.3% of ventricular arrhythmia episodes, and overall mortality was 6%, with no deaths attributed to arrhythmias or the implantation procedure [77].

According to the 2025 Appropriate Use Criteria, combined LP—S-ICD is considered appropriate in patients with LVEF ≥ 50% and an anticipated pacing burden < 40% [62]. It may also be appropriate in selected patients with LVEF < 50% depending on clinical factors such as baseline LVEF, atrial fibrillation burden, and symptomatic bradycardia [62]. However, caution is warranted in patients with reduced LVEF due to potential device interactions. Intermittent pacing may alter QRS and T-wave morphology, potentially resulting in T-wave oversensing by the S-ICD and inappropriate additional shocks [78]. Chronic RV pacing may also promote pacing-induced cardiomyopathy, exacerbating left ventricular dysfunction [77]. In these patients, CRT may be a more appropriate alternative [79].

### 6.4. WiSE CRT: Leadless Left Ventricular Endocardial Pacing

Cardiac resynchronization therapy improves survival and left ventricular (LV) functional status in appropriately selected patients with heart failure [80]. The WiSE CRT system (EBR Systems) was designed to overcome limitations of conventional CRT by providing leadless LV endocardial pacing. The system consists of a small LV endocardial electrode delivered percutaneously to the lateral wall of the LV, along with a subcutaneous ultrasound transmitter and a battery [3]. Pacing stimuli from a co-implanted RV device trigger the transmitter to emit ultrasound energy, which is converted into electrical signals at the LV electrode, thereby enabling synchronized biventricular pacing without a coronary sinus lead [3,81]. In the SOLVE-CRT trial, implantation was successful in 95.5% of patients and was significantly associated with a 16.4% reduction in LV end-systolic volume, demonstrating meaningful reverse remodeling [81]. Emerging feasibility data from 8 patients demonstrated that the LV electrode can provide conduction system pacing when implanted in the LV septum, resulting in significant QRS narrowing [82]. These findings suggest a potential pathway toward fully leadless CRT systems capable of delivering conduction system pacing and more physiologic ventricular activation through direct left bundle branch capture.

Despite these promising results, the WiSE CRT system was associated with a 19.1% higher procedural complication rate than conventional CRT [81]. Reported adverse events included perforation, vascular events, LV electrode embolization, and battery depletion [81]. These findings reflect the technical complexity of the procedure, which should be performed by experienced operators at centers equipped with vascular and cardiothoracic surgical support [83]. As operators become more experienced with this technology, these complications are expected to decrease. Shifting from a retrograde aortic approach to a transseptal approach has significantly reduced vascular access related complications.

## 7. Future Directions

### 7.1. Leadless Biventricular Pacing

The first fully leadless biventricular pacing system combined a Micra LP for RV pacing with the WiSE CRT system for leadless LV endocardial pacing [84]. Carabelli et al. demonstrated the feasibility of this approach in 8 patients, in whom the WiSE transmitter successfully sensed Micra-derived RV activation and delivered synchronized LV pacing [84]. This led to significant QRS narrowing and LVEF recovery at 6 months [84].

Currently, the WiSE-CRT system is the only available approach that allows for a fully leadless CRT system by upgrading patients with a pre-existing LP to CRT [84]. This capability is relevant for patients who subsequently develop heart failure or pacing-induced cardiomyopathy, as well as those who are not candidates for conventional CRT due to unfavorable coronary venous anatomy or risk of infection [81,84].

### 7.2. Conduction System Pacing

Conduction system pacing (CSP) modalities such as left bundle branch area pacing (LBBAP) and His bundle pacing enable more physiologic RV pacing compared to conventional systems [85]. LBBAP has become the predominant CSP approach because of its stable electrical performance, high implantation success, and favorable clinical outcomes [85].

The convergence of CSP and leadless technology represents a promising frontier in cardiac electrophysiology. The LEAP2 study was the first in-human evaluation of chronic implantation of a novel helix-based LP with an extended electrode designed for LBBAP (Aveir CSP LP) [86]. Successful implantation within the interventricular septum was achieved in 18 out of 19 attempts either as a single-chamber ventricular pacemaker (n = 4) or a dual-chamber system (n = 14) [86]. Capture types included left bundle branch pacing, LV septal pacing, and deep septal pacing, with changes in capture characteristics observed in 6 patients over 1 month of follow-up [86]. For dual-chamber systems, i2i communication remained stable during the 1-month follow-up period [86]. The complication-free rate was 68.4%, with 6 patients suffering complications spanning access-site hematoma, device dislodgment, and pericardial effusion [86]. If validated in larger studies, leadless CSP may combine the advantages of leadless systems with the hemodynamic benefits of physiologic pacing, possibly redefining future pacing strategies.

### 7.3. Energy Harvesting and Self-Rechargeable Pacemakers

All current LPs have limited battery capacity, driving interest in self-rechargeable or self-powered devices. Multiple energy-harvesting technologies are currently under development.

Inertia-driven triboelectric nanogenerators (I-TENGs) have demonstrated the ability to harness mechanical energy from body motion, generating sufficient power output (~4.9 μW/cm^3^) to charge a lithium-ion battery in preclinical testing while supporting ventricular pacing and sensing functions in preclinical models [87]. Another capsule-shaped battery-free pacemaker, self-powered by a triboelectric nanogenerator, has been implanted percutaneously in the RV of swine [88]. The device generated approximately 6.0 V of open-circuit voltage and maintained endocardial pacing throughout a 3-week follow-up period [88]. Similarly, flexible multibeam piezoelectric composite thin films integrated into the battery compartment of an LP can harvest energy generated by heartbeat-driven motion, producing voltage outputs sufficient for pacing [89]. Despite being in the early stages of development, these technologies have the potential to extend battery longevity, reduce device replacement procedures, and transform long-term pacing system durability.

## 8. Conclusions

LPs have evolved from niche single-chamber ventricular devices into a versatile and expanding platform capable of atrial pacing, dual-chamber synchronization, and modular integration with S-ICDs, with emerging applications in CRT and CSP. Compared with transvenous systems, LPs are associated with lower rates of chronic complications, device reinterventions, and infections while eliminating lead and pocket-related morbidity. However, they remain associated with a risk of cardiac perforation during implantation. The modular pacing paradigm allows clinicians to tailor hardware to a patient’s pacing requirements and expand functionality over time as clinical needs evolve. While acknowledging the potential energy cost of i2i communication, this philosophy offers a fundamentally less invasive and more flexible framework for long-term rhythm management.

A main limitation of the existing evidence base is the lack of randomized controlled trials directly comparing LPs and TV-PMs, with most comparative data derived from observational studies and registries. Nevertheless, the trajectory of innovation suggests that future cardiac pacing will be increasingly leadless, modular, and physiologically optimized.

## Figures and Tables

**Table 1:** MICRA vs. AVEIR: Device Characteristics and Clinical Outcomes.

Device	MICRA	AVEIR
Manufacturer	Medtronic	Abbott
Introduction	2016	2022
Dimensions	25.9 × 6.7 mm	38 × 6.44 mm
Fixation	Passive nitinol tines	Active helix fixation
Sensor	Accelerometer	Temperature
Projected Battery Longevity	~16.7 years (MICRA VR2)	~17.6 years (AVEIR VR)
Pacing Mode	MICRA VR: VVI/VVIR	AVEIR AR: AAI/AAIR
	MICRA AV: VDD/VDDR	AVEIR VR: VVI/VVIR
		AVEIR DR: DDD/DDDR
Implant Success	99.2% (MICRA IDE)	97.6% (LEADLESS II Phase 2) 98.3% (AVEIR i2i)
Complication rate	4.5% at 5-year follow-up (MICRA VR)	6.8% at 1-year follow up (AVEIR VR) 11.4% at 1-year follow-up (AVEIR DR)
AV Synchrony	Accelerometer-based atrial sensing in MICRA AV only	i2i communication in AVEIR DR
Retrieval	Snare traction collapses tines	Retrieval catheter engages docking feature and unscrews helix

## Data Availability

No new data were created or analyzed in this study.
